# NLRP6 in host defense and intestinal inflammation

**DOI:** 10.1016/j.celrep.2021.109043

**Published:** 2021-04-27

**Authors:** K. Venuprasad, Arianne L. Theiss

**Affiliations:** 1 Department of Internal Medicine, UT Southwestern Medical Center, Dallas, TX 75390, USA; 2 Department of Immunology, UT Southwestern Medical Center, Dallas, TX 75390, USA; 3 Harold C. Simmons Comprehensive Cancer Center, University of Texas Southwestern Medical Center, Dallas, TX 75390, USA; 4 Division of Gastroenterology and Hepatology, School of Medicine at the Anschutz Medical Campus, University of Colorado, Aurora, CO 80045, USA

## Abstract

NLRP6 is a member of the NLR (nucleotide-oligomerization domain-like receptor) family of proteins that recognize pathogen-derived factors and damage-associated molecular patterns in the cytosol. The function of NLRP6 has been attributed to the maintenance of epithelial integrity and host defense against microbial infections. Under some physiological conditions, NLRP6 forms a complex with ASC and caspase-1 or caspase-11 to form an inflammasome complex cleaving pro-interleukin-1β(IL-1β) and IL-18 into their biologically active forms. Here, we summarize recent advances in the understanding of the mechanisms of activation of the NLRP6 inflammasome and discuss its relevance to human disease.

## INTRODUCTION

Inflammation is a host response against microbial infections and tissue damage to limit harm to the body. Inflammation is initiated following the sensing of microbial components and signs of acute damage or disturbances of the steady state (Henao-Mejia et al., 2014; Medzhitov, 2008). Several mechanisms have evolved to distinguish between homeostasis and threats to the host, which include pattern recognition receptors (PRRs). These receptors recognize distinct pathogen-associated molecular patterns (PAMPs) that are predominantly found in microbes and hence allow the sensing of pathogens in tissues (Medzhitov and Janeway, 1997). PAMPs are located either in the cytosol, on the plasma membrane, or in endosomal compartments. Prototypic families of PRRs include the Toll-like receptors (TLRs), C-type lectin receptors (CTLs), RIG-I-like receptors (RLRs), and nucleotide-oligomerization domain (NOD)-like receptors (NLRs) (Kanneganti, 2010; Medzhitov, 2008; Takeuchi and Akira, 2010). In the presence of a specific microbial ligand, these PRRs trigger a downstream signaling cascade that leads to the activation of transcription factors and to the production of pro-inflammatory cytokines. These cytokines orchestrate the switch from tissue homeostasis to a state of inflammation that is aimed at removing the trigger of PRR signaling and restoring normal tissue function (Davis et al., 2011; Kanneganti, 2010; Medzhitov, 2008). In addition to PAMPs, PRRs can recognize host-derived signals, called damage-associated molecular patterns (DAMPs), which are released as a result of perturbations of tissue homeostasis caused by microbial or non-microbial insults (Matzinger, 1994).

NLRs are a group of cytosolic sensors of both PAMPs and DAMPs that are activated by both endogenous and exogenous triggers (Bryant and Fitzgerald, 2009; Strowig et al., 2012). They share a similar domain structure consisting of a central nucleotide-binding and oligomerization (NACHT) domain, commonly flanked by C-terminal leucine-rich repeats (LRRs) and N-terminal caspase recruitment domains (CARDs) or pyrin domains (PYDs). LRRs are believed to function in ligand sensing and autoregulation, whereas CARDs and PYDs mediate homotypic protein-protein interactions for downstream signaling (Henao-Mejia et al., 2014). Based on the N-terminal domains, NLRs are divided into four distinct subfamilies: the NLRA (CIITA), NLRB (NIAP), NLRC (NOD1, NOD2, NLRC3, NLRC4, NLRC5), and NLRP (NLRP1–14) (Ting et al., 2008). Upon recognition of PAMPs or DAMPs, some of the NLRs form a multimeric protein complex called the inflammasome (Kanneganti, 2010; Schroder and Tschopp, 2010). The core function of the inflammasome is the recruitment and activation of pro-inflammatory caspases (caspase-1 or -11), resulting in the cleavage of interleukin-1β(IL-1β) and IL-18 precursors into their bioactive forms. IL-1β and IL-18 are potent pro-inflammatory cytokines that exert a wide range of functions in inflammation and in maintenance of tissue integrity (Henao-Mejia et al., 2014; Medzhitov, 2008; Schroder and Tschopp, 2010). Emerging evidence suggests cell-type- or tissue-specific NLRP6 functions (Table 1) with a critical role for NLRP6 in host defense against microbial infection and intestinal inflammation. In this review, we summarize recent advances in the mechanism of activation of NLRP6, its role in the regulation of gut inflammation, and the controversies in the modulation of microbiota.

### NLRP6 inflammasome

NLRP6 was originally called PYPAF5 and was expressed predominantly in mucosal tissues that are constantly exposed to microbial components. NLRP6 is expressed by epithelial cells, fibroblasts, granulocytes, dendritic cells, CD4 and CD8 T cells, and macrophages (Elinav et al., 2011). The mechanisms by which NLRP6 expression are regulated remain largely unclear. NLRP6 promoter analysis has shown the presence of peroxisome-proliferator-activated receptor-γ (PPAR-γ) and retinoid X receptor-α (RXR-α) binding motifs (Kempster et al., 2011). Accordingly, NLRP6 expression was enhanced in human and mouse colon epithelial cells treated with rosiglitazone, a PPAR-γ agonist. *NLRP6* mRNA expression was also shown to be induced by the encephalomyocarditis virus (EMCV), polyinosinic:polycytidylic acid (poly(I:C)), and interferon-α (IFN-α) in mouse fibroblasts (Wang et al., 2015). Furthermore, the type I IFN pathway was shown to be essential for the induction of NLRP6 expression in bone-marrow-derived macrophages (BMDMs) (Hara et al., 2018). These data suggest the involvement of microbial and metabolic signals in the regulation of NLRP6 expression.

The initial studies of co-expression of NLRP6 and ASC resulted in caspase-1 activation, which led to the concept that NLRP6 forms an inflammasome like other members of the NLR family (Levy et al., 2015). The *in vivo* evidence for NLRP6 inflammasome formation was provided by the demonstration that *Nlrp6*^−/−^ mice have reduced serum IL-18 levels under steady-state conditions and after dextran sulfate sodium (DSS)-induced colitis compared with that of wild-type (WT) controls (Elinav et al., 2011). Furthermore, Levy et al. (2015) demonstrated that NLRP6 co-localizes with ASC in intestinal cells to form an inflammasome.

**Figure 1.**
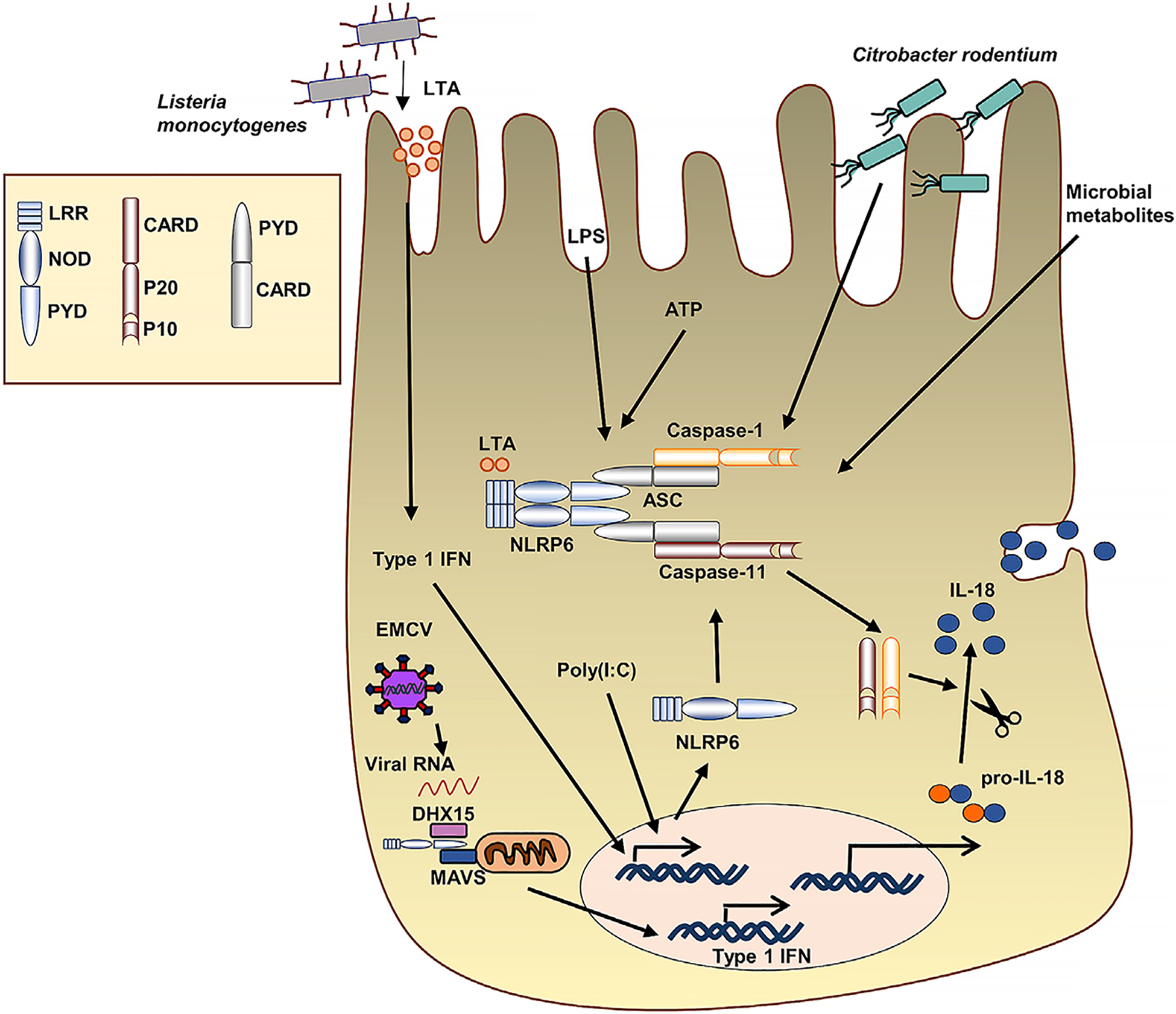
Activation of the NLRP6 inflammasome following microbial infection LTA, a component of *Listeria*, induces type I IFN and upregulates NLRP6. Similarly, viral RNA and poly(I:C) induce Nlrp6 expression. NLRP6 recruits ASC and pro-caspase-1/caspase-11 to form the NLRP6 inflammasome. Nlrp6 can also be activated by LPS + ATP as well as C. *rodentium* infection. NLRP6 inflammasome activates caspase-1, which cleaves pro-IL-18 and pro-IL-1β into their active forms that are then secreted by exocytosis.

Recently, we and others have reported that the NLRP6 inflam- masome is activated during bacterial infections (Elinav et al., 2011; Hara et al., 2018; Mukherjee et al., 2020). We showed that infection of *Nlrp6*^−/−^ mice with *Citrobacter rodentium* resulted in reduced caspase-1 activation and IL-18 processing (Mukherjee et al., 2020). Consistently, it was demonstrated that *Nlrp6*^−/−^ BMDMs showed reduced caspase-1 activity and IL-1β secretion compared with WT BMDMs infected with *Staphylococcus aureus* (Ghimire et al., 2018). NLRP6 co-localizes with ASC in BMDMs infected with *S. aureus* (Ghimire et al., 2018). Similarly, *Listeria monocytogenes* activates the NLRP6 inflammasome (Hara et al., 2018). Interestingly, lipoteichoic acid (LTA) from *L. monocytogenes* upregulates the expression of NLRP6 and caspase-11 via type I IFN signaling. LTA also binds to NLRP6 and activates the inflammasome via ASC-caspase-11 and -caspase-1 (Figure 1). This growing evidence from independent laboratories suggests that NLRP6 assembles into an inflammasome. However, further investigations are required to determine how *S. aureus* and *C. rodentium* activate NLRP6. It is possible that either the cell membrane components or toxins from these bacteria activate type I IFN signaling, similar to *Listeria*. In fact, type I IFN signaling induced by *S. aureus* was shown to be dependent on the virulence factor protein A, specifically the Xr domain, which is a short sequence repeat region encoded by variable numbers of 24-bp repeated DNA sequences (Martin et al., 2009). Additionally, the *C. rodentium* type III secretion effector NleB modulates the type I IFN response (Gao et al., 2016).

### Activation of NLRP6 inflammasome

In the absence of inflammatory stimuli, inflammasome activation is prevented by the closed conformation of the LRR and NACHT domains of NLRs (Hu et al., 2013). Cryoelectron microscopy (cryo-EM) and structure-based investigations have revealed that the assembly of the NLRP6 inflammasome involves two nucleation-induced polymerization steps (Shen et al., 2019). In the first step, nucleation of ASC filaments by oligomerized NLRP6 through a PYD-PYD interaction leads to polymerization of ASC. In the second step, the polymerized ASC nucleates caspase-1 filaments via a CARD-CARD interaction, leading to caspase-1 activation. Activated caspase-1, in turn, facilitates the maturation of pro-IL-1β and pro-IL-18 (Lamkanfi and Dixit, 2014; Ruan et al., 2018).

The cryo-EM and crystal structure of NLRP6 has recently been solved (Shen et al., 2019). The authors purified full-length NLRP6 (NLRP6^FL^), NLRP6 containing only the PYD (NLRP6^PYD^), and NLRP6 containing both the PYD and NBD (NLRP6^PYD+NBD^) and tested their ability to induce ASC^PYD^ polymerization. All NLRP6 constructs were able to promote ASC^PYD^ polymerization, but to a different extent. NLRP6^PYD+NBD^ was the strongest nucleator of ASC^PYD^ polymerization (Hill coefficient of 0.33), followed by NLRP6^PYD^ (Hill coefficient of 0.71). NLRP6^FL^ was found to be the weakest nucleator among all, with the highest dependence on concentration. It also showed the highest Hill coefficient of 4.2 in promoting ASC polymerization. The low Hill coefficients for NLRP6^PYD^ and NLRP6^PYD+NBD^ and their high ability to polymerize ASC compared with NLRP6^FL^ might be related to the presumed autoinhibited conformation of the FL protein. The NLRP6^PYD^ filaments possess a hollow cylindrical architecture that assembles through a right-handed helix, forming multiple layers. The NLRP6-PYD makes a filamentous structure that provides the stage for recruitment of the ASC-PYD and oligomerization through NLRP6-PYD:ASC-PYD interaction. The polymerized ASC nucleates caspase-1 filaments via a CARD-CARD interaction, leading to caspase-1 activation. Activated casase-1, in turn, facilitates the maturation of pro-IL-1β and pro-IL-18.

**Figure 2.**
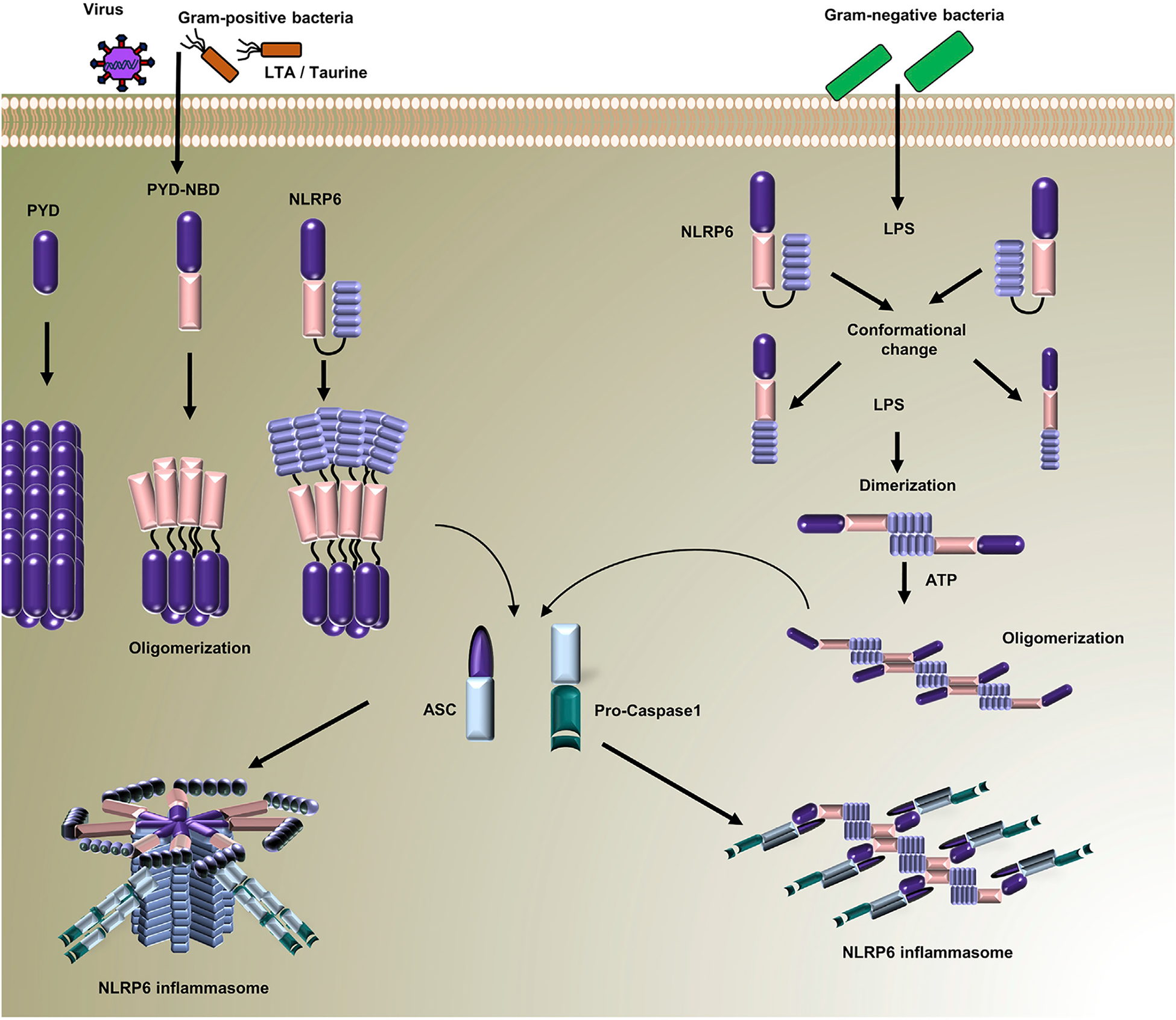
A model for NLRP6 Inflammasome assembly during microbial infections Under resting conditions, NLRP6 remains in an auto-inhibited form. Infections by virus or Gram-positive bacteria activate NLRP6, resulting in its oligomerization through NBDs and PYDs in which PYD filamentous core surrounded by NBD and LRR domain. PYD filaments provide the platform for ASC recruitment and oligomerization through PYD-PYD interactions. The CARD in ASC then oligomerizes and recruits caspase-1, driving caspase-1 dimerization and activation. By contrast, during Gram-negative bacterial infections, LPS directly binds to LRR domain of NLRP6 and induces a conformational change, resulting in its linear dimerization. In the presence of ATP, the NLRP6 homodimer further self-assembles into even larger oligomers, providing a linear molecular platform for the recruitment of ASC and caspase-1, which then assemble into the inflammasome.

Leng et al. (2020), however, provided an alternate mechanism for activation of the NLRP6 inflammasome. They demonstrated that NLRP6 is activated by lipopolysaccharide (LPS) and ATP, a process similar to NLRP3 activation. LPS directly binds to the LRR of the NLRP6 monomer and initiates its dimerization. In this homodimer model, the major interface for dimerization was formed by the interaction of two LRR domains of NLRP6 in an antiparallel manner. However, LPS-induced NLRP6 oligomerization could not go beyond a dimer, but LPS along with ATP triggered the formation of higher oligomeric NLRP6 in a linear arrangement. This provides a novel linear platform for the recruitment of ASC and inflammasome activation (Leng et al., 2020). The major difference between the two models is the ring-like inflammasome arrangement in which NLRP6^PYD^ is surrounded by the NBD and LRR domain (Shen et al., 2019) versus the linear arrangement of NLRP6 oligomerization following LPS and ATP stimulation (Leng et al., 2020). How these two different models operate *in vivo* during an inflammatory response remains elusive and needs further investigation. It is possible that Gram-positive and Gram-negative bacteria elicit these mechanisms in differing manners (Figure 2).

Another means of inflammasome activation is via ubiquitination, which is a form of post-translational modification in which protein substrates are conjugated to ubiquitin by E3 ubiquitin ligases (Venuprasad et al., 2006). Ubiquitin contains seven Lys (K) residues through which it can form ubiquitin chains, but the ubiquitin linkage generally occurs through K48 or K63. K48-linked ubiquitination leads to protein degradation, whereas K63-linked ubiquitination leads to non-proteasomal modifications such as protein complex formations (Venuprasad, 2010). Protein ubiquitination is also highly dynamic and subjected to deubiquitination by deubiquitinating enzymes (DUBs) (Venuprasad et al., 2015). The initial evidence for ubiquitination in inflammasome activation came from inhibition of deubiquitination with the isopeptidase inhibitor G5, where the activation of NLPR3 inflammasome was inhibited. The DUB BRCC3 was shown to deubiquitinate the LRR region of NLRP3 prior to NLRP3 assembly and activation. We have recently reported that NLRP6 undergoes K63-linked ubiquitination, which promotes its association with ASC (Mukherjee et al., 2020). The mechanism by which K63-linked ubiquitination promotes NLRP6 inflammasome activation remains unclear. However, it is possible that ligands binding to NLRP6 could promote K63-linked ubiquitination resulting in a conformational change. This may allow NLRP6 to overcome the autoinhibition, leading to recruitment of ASC and inflammasome activation. Alternatively, it is possible that K63-linked ubiquitination promotes oligomerization of NLRP6.

### NLRP6 in the regulation of microbial infections

Inflammasomes play a critical role in the innate immune response against microbial infections (Anand et al., 2011; Hara et al., 2018; Mukherjee et al., 2020; Vladimer et al., 2013). Anand et al. (2012) demonstrated that deletion of NLRP6 resulted in enhanced bacterial clearance and improved survival in *Nlrp6*^−/−^ mice infected with *Listeria*, *Salmonella*, and *Escherichia coli*. This protection was attributed to enhanced nuclear factor κB (NF-κB) and mitogen-activated protein kinase (MAPK) activity. Interestingly, there was no differences in the level of IL-1β or caspase-1 activation in *Nlrp6*^−/−^ mice, suggesting an inflammasome-independent mechanism. Similarly, NLRP6 acted as a negative regulator of pulmonary host defense during Gram-positive bacteria (*S. aureus*) infection of the lungs (Ghimire et al., 2018). By contrast, in a murine model of *C.-rodentium*-induced colitis, NLRP6 deficiency resulted in impaired host defense. Intestines from *Nlrp6*^−/−^ mice were extensively colonized with *C. rodentium* and displayed extensive mucosal ulceration, edema, and hyperplasia compared with WT mice (Ghimire et al., 2018; Wlodarska et al., 2014). Consistent with Anand et al. (2012), Hara et al. (2018) recently demonstrated that Nlrp6 deficiency resulted in increased clearance of *Listeria*. LTA from *Listeria* binds to NLRP6 and activates the NLRP6 inflammasome via ASC to regulate host defense. Interestingly, NLRP6 activated both caspase-11 and caspase-1 upon binding of LTA or *Listeria* for processing of IL-1β and IL-18. Upon infection with *Listeria*, *Nlrp6*^−/−^ mice showed reduced bacterial burdens compared with WT mice. This protection was abolished when these mice received recombinant IL-18, but not IL-1β, suggesting that the NLRP6 inflammasome exacerbates *Listeria* infection via IL-18 (Hara et al., 2018).

In addition to the role of NLRP6 in bacterial infections, it also plays a crucial role in viral infections, as shown by Wang et al. (2015). Both WT and *Nlrp6*^−/−^ mice exhibited no difference in survival when infected with EMCV. However, *Nlrp6*^−/−^ mice had higher viral loads in the intestine, suggesting that NLRP6 may play an important role in viral clearance from the intestine. Interestingly, *Nlrp6*^−/−^ mice displayed increased susceptibility to EMCV when administered orally; similar results were obtained for oral infection with murine norovirus (Wang et al., 2015). Mechanistically, NLRP6 associates with the Dhx15 helicase to form a viral sensing complex that recognizes cytosolic long double-stranded DNA (dsRNA) and activated mitochondrial antiviral signaling proteins (MAVS) to initiate the antiviral response (Wang et al., 2015).

Thus, NLRP6 plays a protective role in the host against bacterial and viral infections in the intestine, where it is highly expressed. However, in systemic and pulmonary infections, NLRP6 expression appears to have negative effects (Ghimire et al., 2020). It is possible that in bacterial infections, where myeloid cells are most important, NLRP6 seems to trigger destructive inflammation; however, during enteritis, involving non-hematopoietic cells such as intestinal epithelial cells, the NLRP6-mediated response is protective. Nonetheless, more studies are necessary to further define the differential role of NLRP6 in viral, fungal, and bacterial infections.

### NLRP6 in colonic inflammation

NLRP6 is predominantly expressed in the small and large intestine, especially in enterocytes, colonic goblet cells, and myofibroblasts (Normand et al., 2011), suggesting a key role for NLRP6 in the maintenance of gut homeostasis. Deletion of *Nlrp6* aggravates DSS-induced colitis or colitis-associated tumor growth due to deregulated regeneration and proliferation of intestinal epithelial cells (Normand et al., 2011). Since an altered microbiota play a critical role in colonic inflammation, Elinav et al. (2011) performed 16S ribosomal RNA analysis of fecal samples and found a microbiota shift toward a higher abundance of the bacterial family Prevotellaceae and phyla TM7 in *Nlrp6*^−/−^ mice compared with WT mice. Interestingly, co-housing of *Nlrp6*^−/−^ mice transferred microbiota to WT mice, resulting in enhanced susceptibility of WT mice to colitis (Elinav et al., 2011). However, Mamantopoulos et al. (2017) did not observe any difference in microbiota composition between WT and *Nlrp6*^−/−^ mice. Porphyromonadaceae and Bacteroidaceae, but not Prevotellaceae, were differentially represented in these mice. These differences were due to mother and cage covariates rather than Nlrp6 deficiency. In support of this finding, Lemire et al. (2017) also found that Nlrp6 did not impact gut microbiota composition by using littermate *Nlrp6*^−/−^ and *Nlrp6*^+/+^ mice, suggesting that Nlrp6 does not regulate microbiota composition. On the contrary, Seregin et al. (2017c) observed significant differences in microbiota composition between *Nlrp6*^−/−^*/IL-10*^−/−^ and *Nlrp6*^+/+^*/IL-10*^−/−^ littermate control mice, supporting the notion that Nlrp6 influences the composition of gut microbiota. One possible explanation for these discrepancies are non-genetic factors such as familial transmission and stochastic events. In support of this possibility, Gálvez et al. (2017) reported that microbiota composition varies greatly within the segregated colonies of the same genotype, even within the same facility. Furthermore, the presence of specific pathobiont within a facility could be attributed to genotype-linked microbiota composition. Therefore, it is possible that the presence of a specific pathobiont in one facility, but not in the other, might contribute to these discrepant results.

**Figure 3.**
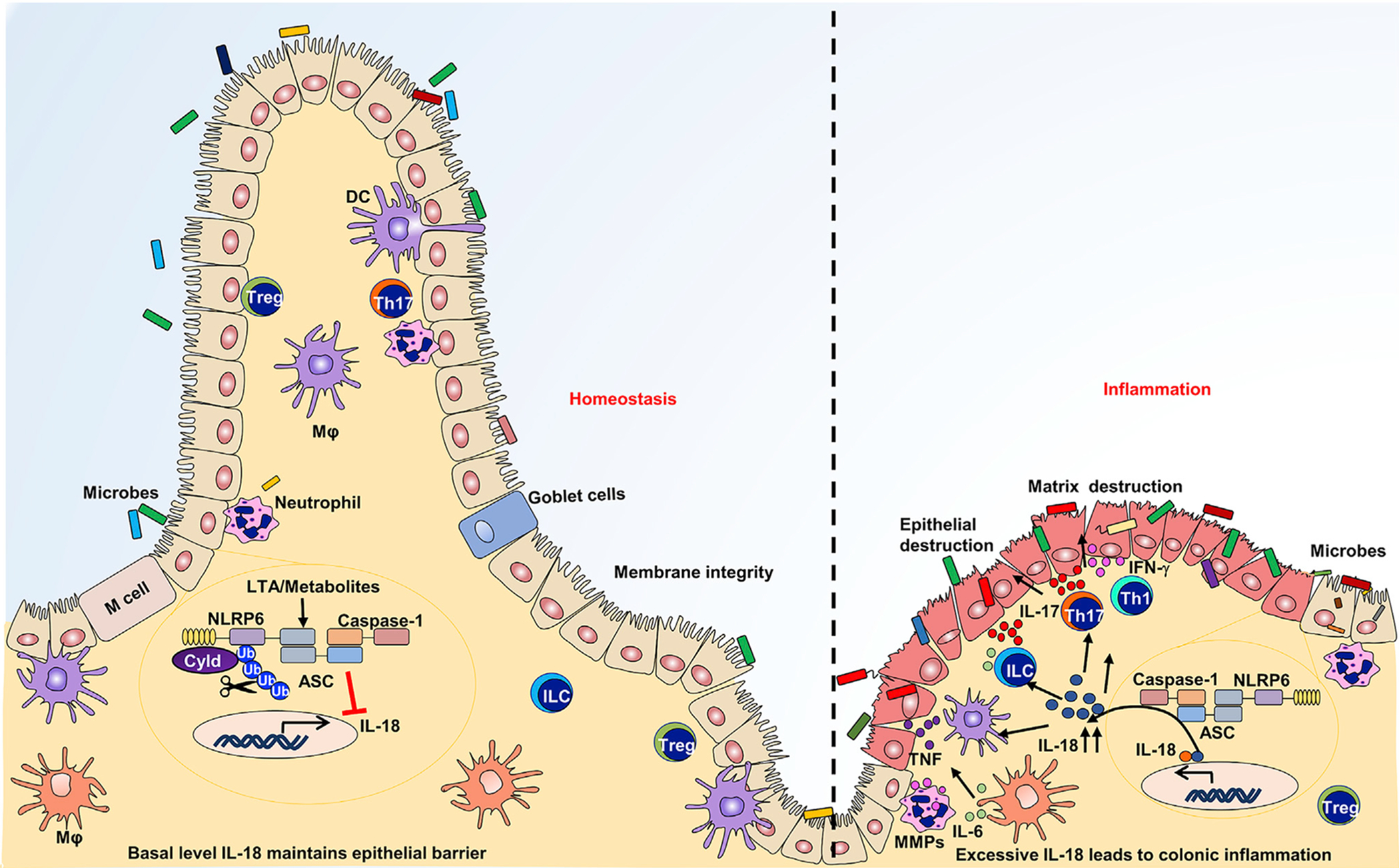
Regulation of optimal NLRP6 inflammasome activation and abundance of IL-18 in the colonic mucosa Microbial components and metabolites induce the formation of the NLRP6 inflammasome. CYLD prevents sustained inflammasome activation via its deubiquitination. In UC patients, reduced *CYLD* expression leads to excessive NLRP6 inflammasome activation, resulting in elevated levels of IL-18. Excessive IL-18 amplifies inflammation by promoting IFN-γ, TNF-α, IL-17, and IL-6.

NLRP6 is also linked to epithelial integrity through the regulation of goblet cell function and secretion of antimicrobial peptides (Wlodarska et al., 2014). It was shown that NLRP6 is essential for homeostatic mucin secretion by goblet cells. *Nlrp6*^−/−^ mice exhibited reduced autophagy and hyperplasia of goblet cells and a failure to exocytose mucin granules. This resulted in a thin mucus layer over the epithelium, leading to increased susceptibility to enteric infections (Wlodarska et al., 2014). Our group recently demonstrated that CYLD, a DUB, negatively regulates the NLRP6 inflammasome and prevents excessive IL-18 levels in the colonic mucosa (Mukherjee et al., 2020). IL-18 has both a protective and detrimental role in colonic inflammation. Increased expression and bioactivity of IL-18 correlate with disease severity in inflammatory bowel disease (IBD) patients (Monteleone et al., 1999; Pizarro et al., 1999). Also, genome-wide association studies have revealed an association of variants within the IL-18R1-IL-18RAP locus with IBD (Barrett et al., 2008; Hedl et al., 2014; Imielinski et al., 2009). In line with these data, conditional deletion of IL-18 in intestinal epithelial cells or myeloid cells results in decreased severity of intestinal inflammation (Nowarski et al., 2015). However, complete loss of IL-18, IL-18R, or components of the inflammasome predisposes mice to increased epithelial damage and potentiates colonic tumor growth (Salcedo et al., 2010; Takagi et al., 2003; Zaki et al., 2010). This suggests that a basal level of IL-18 in the colonic mucosa is required to maintain barrier integrity, whereas elevated levels of IL-18 promote inflammation and intestinal damage. Our results show that Cyld deficiency resulted in severe colitis, which was associated with an increased level of NLRP6 inflammasome activity and IL-18 in the colonic mucosa. Furthermore, neutralization of IL-18 attenuates colonic inflammation in *Cyld*^−/−^ mice (Mukherjee et al., 2020). These data suggest that NLRP6 function is tightly regulated in the colonic mucosa to prevent pathogenic inflammation (Figure 3). Further detailed investigation is essential to fully understand the dichotomy of protective/pathogenic inflammation mediated by NLRP6.

### NLRP6 in human diseases

Consistent with the mouse model data, the transcriptomic analysis showed abundant expression of NLRP6 in the human intestine, suggesting that NLRP6 has an important role in maintaining gut homeostasis in humans (Gremel et al., 2015). However, our recent data showed no significant change in the expression of NLRP6 in human ulcerative colitis (UC) patients compared with healthy controls (Mukherjee et al., 2020). This is consistent with another report showing insignificant *NLRP6* alterations in mRNA expression in IBD patients (Alipour et al., 2016). We and others have demonstrated that the expression of *CYLD*, which deubiquitinates NLRP6, is downregulated in UC patients (Costello et al., 2005; Mukherjee et al., 2020). Furthermore, the levels of *CYLD* expression are negatively correlated with *IL-18* expression in the colonic mucosa of UC patients (Mukherjee et al., 2020). This suggests that the regulatory mechanisms inhibiting excessive activation of NLRP6-mediated inflammation are defective in patients.

Colonic inflammation increases the risk of developing colon cancer among IBD patients (Grivennikov et al., 2010). Although the expression of NLRP6 is essential to prevent colorectal cancer in murine models, gene expression analysis of colorectal cancer patients shows no change in the expression of *NLRP6* (Liu et al., 2015). It is possible that the mechanisms that regulate NLRP6 in colon cancer could be defective and require further investigation. Since defects in CYLD expression or mutations have been reported in colon cancer (AACR Project GENIE Consortium, 2017; Hellerbrand et al., 2007), the involvement of the CYLD-NLRP6 pathway needs to be investigated. Such studies could lead to novel therapeutic strategies to potentially target NLRP6 in colon cancer. NLRP6 could have a regulatory function in human lung infections, as suggested by Ghimire et al. (2018), who showed an increased expression of NLRP6 in neutrophils, macrophages, and epithelial cells in the lungs obtained from pneumonia patients. Upregulation of NLRP6 and IL-18 was also reported in adipose tissues obtained from NASH patients with portal fibrosis compared with that from control patients, suggesting a role of NLRP6 in liver disease (Henao-Mejia et al., 2012; Kanda et al., 2020). In another study of patients undergoing endodontic microsurgery, analysis of tissues associated with apical periodontitis revealed higher expression of NLRP6 (Lu et al., 2019). Similarly, increased NLRP6 was reported in the inflamed human dental pulp tissue of pulpitis patients (Tian et al., 2020). An anti-inflammatory role of NLRP6 has been reported in rheumatoid arthritis patients in which NLRP6 was found to be downregulated in synovial tissues and fibroblast-like synoviocytes (FLSs) in rheumatoid arthritis patients compared with osteoarthritis patients (Lin and Luo, 2017). Intriguingly, in a genome-wide association study, a single-nucleotide polymorphism in NLRP6 has been linked to mean platelet volume, suggesting a potential involvement of this NLR in platelet function (Gieger et al., 2011). Thus, a clear understanding of the role of NLRP6 in human disease is currently lacking, which is essential to target NLPRP6 effectively.

## Concluding remarks

NLRP6 exhibits diverse functions in the regulation of responses against pathogenic infections and gut homeostasis. Conflicting observations in different studies suggest that NLRP6 harnesses context-reliant inflammasome-dependent and -independent functions. Similarly, NLRP6 seems to have both protective and detrimental effects against microbial pathogens in the intestine and other mucosal surfaces. Studies involving deletion of NLRP6 in specific cell compartments, such as myeloid cells, epithelial cells, or lymphocytes, could provide more conclusive findings.

Since NLRP6 recruits both caspase-1 and caspase-11 to form an inflammasome, future biophysical and biochemical studies are essential to understand how these caspases are recruited during NLRP6 inflammasome assembly. Similarly, how NLRP6 function and stability are regulated remain to be investigated. It is likely that post-translational modifications such as phosphorylation, ubiquitination, and sumoylation could modulate its function. Also, complexity might exist in the upstream regulators of NLRP6. Furthermore, the discrepancies regarding the role of NLRP6 in the regulation of gut microbiota need careful evaluation. Finally, the majority of the functions of NLRP6 are currently studied in mouse models, and exploring the full spectrum of cellular functions of NLRP6 in humans could lead to novel therapeutic strategies for human diseases.

## Figures and Tables

**Table 1.** NLRP6 functions across different tissues and cell types

Tissue	Cell type	NLRP6 function
Intestine	goblet cells	mucus secretion (involved in the prevention of gut microbiota dysbiosis) (Birchenough et al., 2016; Wlodarska et al., 2014) autophagosome formation (Wlodarska et al., 2014)
	epithelial cells	epithelial restitution during colitis/injury; protection against colitis (Chen et al., 2011; Elinav et al., 2011; Normand et al., 2011; Seregin et al., 2017b) antimicrobial peptide secretion (involved in the prevention of gut microbiota dysbiosis) (Levy et al., 2015) response to viral infection (Wang et al., 2015) autophagosome formation (Wlodarska et al., 2014)
	hematopoietic cells Ly6C^hi^ inflammatory monocytes and neutrophils	protection against colitis-associated tumorigenesis (Chen et al., 2011; Normand et al., 2011) activates IL-18-induced TNF-α production to ameliorate intestinal inflammation (Seregin et al., 2017a)
	macrophages and neutrophils	suppression of TLR-induced NF-κB and MAPK signaling, decreasing production of TNF-α and IL-6, dampening inflammatory response to bacterial (*L. monocytogenes*, *S. typhimurium*, and *E. coli*) infection (Anand et al., 2012)
Liver	hepatic stellate cells not defined	activation of pro-fibrotic effects (Zhu et al., 2018) negatively regulates NAFLD/NASH progression and metabolic syndrome via modulation of the gut microbiota (Henao-Mejia et al., 2012) protection against liver damage after allogeneic hematopoietic stem cell transplantation (Li et al., 2019) protection against steatosis, inflammation, and fibrosis during alcoholic hepatitis (Ji et al., 2020) mediator of hepatic response to *Schistosoma mansoni* (*S. mansoni*) infection (Sanches et al., 2020)
Lung	neutrophils	negative regulator of response to bacterial (*S. aureus)* infection (Ghimire et al., 2018) critical for host survival and neutrophil function to clear bacterial (*Klebsiella pneumonia* [*K. pneumonia*]) infection (Cai et al., 2020)
Kidney	tubular epithelial cells	protection against acute kidney injury (Valiño-Rivas et al., 2020)
Brain	not defined	activates autophagy and inflammation, leading to brain injury during intracerebral hemorrhage (Nie et al., 2020; Wang et al., 2017; Xiao et al., 2020) pro-inflammatory effect in cerebral ischemia/reperfusion (I/R) injury (Meng et al., 2019; Zhang et al., 2020)
Immune	naive T cells	promotes survival and differentiation into T helper 1 (Th) cells (Radulovic et al., 2019)
Joint	FLSs	dampens pro-inflammatory cytokine production and NF-κB in rheumatoid arthritis FLS (Lin and Luo, 2017)
